# Supplementary figures and images for: Velvet Family Protein FpVelB Affects Virulence in Association with Secondary Metabolism in Fusarium pseudograminearum
Source: Cells. 2024 May 30;13(11):950. doi: 10.3390/cells13110950 (PMC11171821; doi:10.3390/cells13110950)

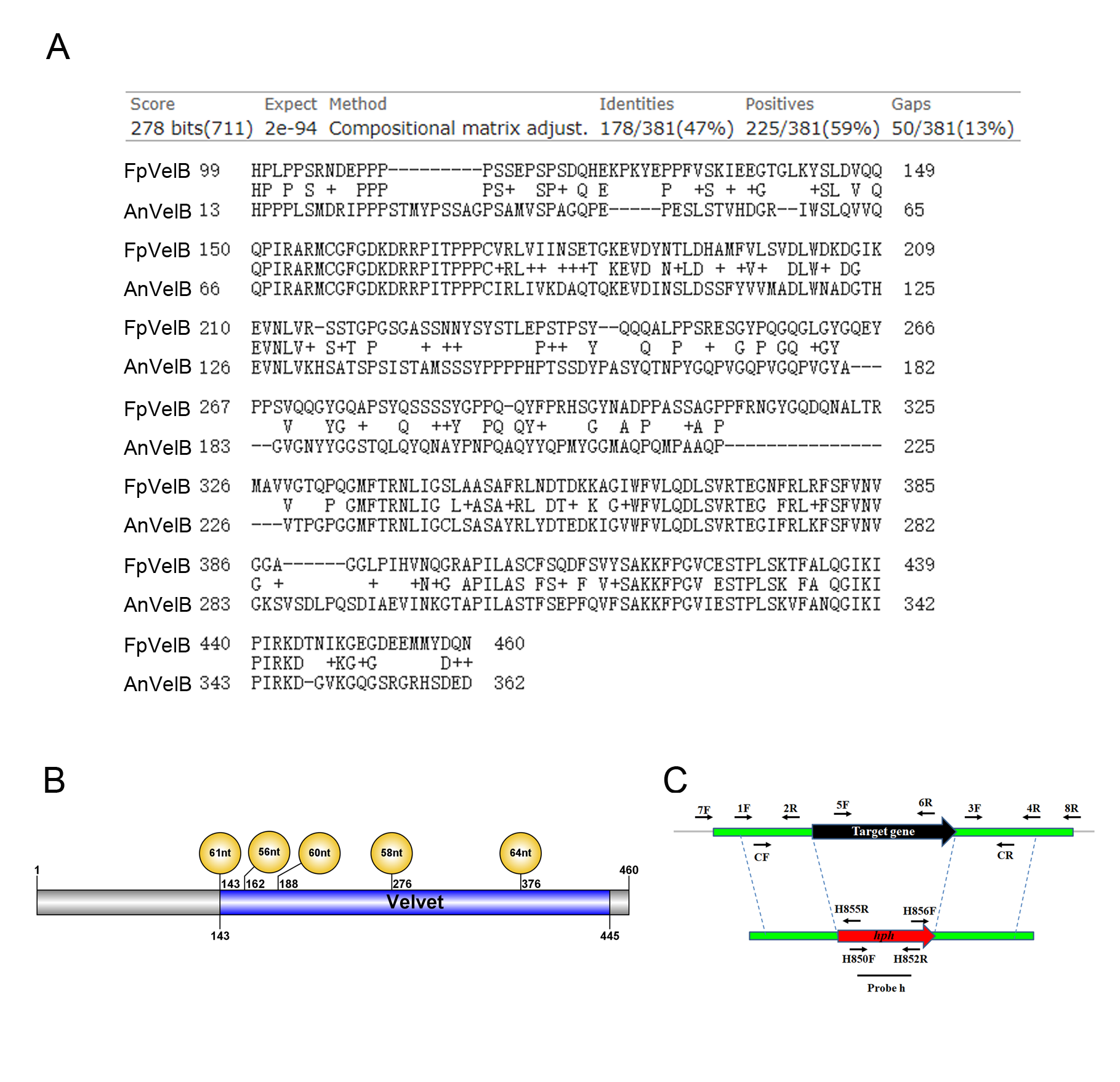

Supplement: Supplementary file 1 [file cells-13-00950-s001.zip › Figure S1.tif]

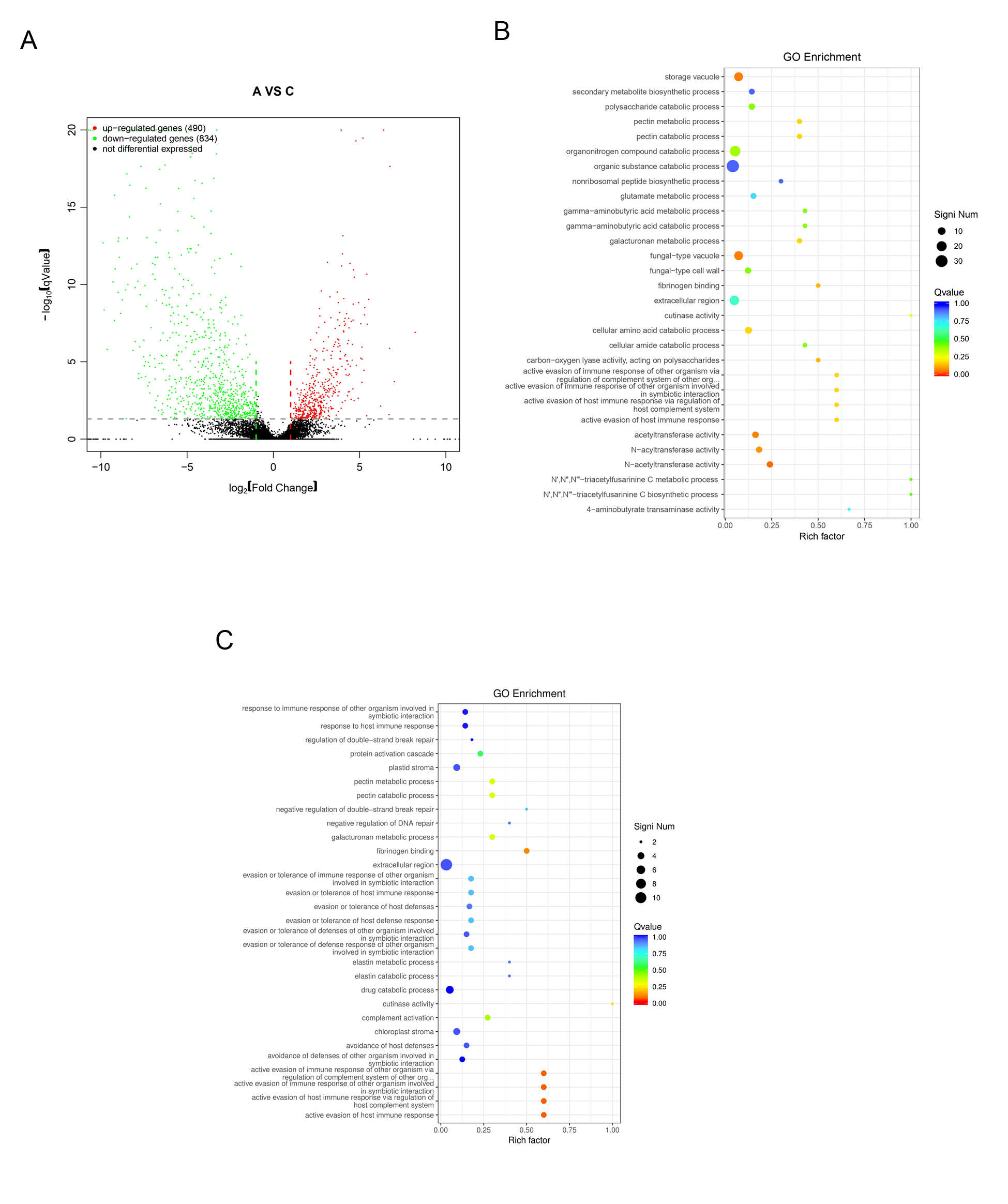

Supplement: Supplementary file 1 [file cells-13-00950-s001.zip › Figure S2.tif]
